# Large-scale production of lentiviral vectors using multilayer cell factories

**DOI:** 10.14440/jbm.2018.236

**Published:** 2018-04-10

**Authors:** Nathan Rout-Pitt, Alexandra McCarron, Chantelle McIntyre, David Parsons, Martin Donnelley

**Affiliations:** Department of Respiratory and Sleep Medicine, Women’s and Children’s Hospital, 72 King William Road, North Adelaide SA 5006, Australia; Robinson Research Institute, Adelaide SA 5000, Australia; Adelaide Medical School, University of Adelaide, Adelaide SA 5005, Australia

**Keywords:** calcium phosphate, cell factories, lentiviral vector, Mustang Q, ultracentrifugation

## Abstract

Lentiviral-mediated gene therapy has been proposed for the treatment of a range of diseases, and due to its genome integration properties, it offers the potential for long-lasting benefit from a once-off treatment. Production methods for pre-clinical studies in animal models, and ultimately for human clinical trials, must be capable of producing large quantities of high-quality lentiviral vector in an efficient and cost-effective manner. We report here a medium-scale method (from 1.5 L to 6 L of vector supernatant) for lentiviral vector production in adherent cell cultures using the NUNC™ EasyFill™ Cell Factory™ from Thermo Fisher Scientific. Downstream purification uses a Mustang Q XT5 anion exchange capsule from Pall, and an ultracentrifugation step to concentrate the vector. This method is capable of producing lentiviral vector with concentrated titres of 10^8^–10^9^ TU/ml, with reduced manual handling compared to single monolayer flask methods.

## BACKGROUND

Gene-addition therapy is a promising therapeutic approach with the potential to treat or cure a range of genetic and acquired diseases by introducing a working copy of the appropriate gene into affected target cells. Lentiviral (LV) vectors are advantageous for therapeutic development because they transduce dividing and non-dividing cells, can be pseudotyped to target specific cell types by altering surface receptor recognition elements, and show high levels of gene transfer efficiency [1].

Phase II trials for metachromatic leukodystrophy [2], Wiskott-Aldrich syndrome [3,4], and Parkinson’s disease [5] have reported therapeutically-effective outcomes using LV vectors. Similarly, gene therapies for β-globinopathies (sickle-cell anemia and β-thalassemia) have survived the rigors of pre-clinical safety and efficacy testing [6]; with long-term, multi-institutional, phase III/IV studies now underway in Europe and the USA [7]. The VSV-G pseudotyped, LV vector described here [8] has been applied to gene therapy studies for Sanfilippo syndrome, Sly syndrome, methylmalonic aciduria, and cystic fibrosis [9-12]. Our primary interest is treating the airway disease in cystic fibrosis using a HIV-1 LV vector to introduce a working copy of the cystic fibrosis transmembrane conductance regulator (CFTR) gene into airway epithelial cells [11].

Producing sufficient quantities of LV vector for use in pre-clinical animal studies and clinical trials has proven challenging. Typically, LVs are produced using either stable packaging cell lines or by the transient transfection of adherent human embryonic kidney (HEK) 293T cells [13]. Ultimately, a stable packaging cell line would be ideal for up-scaling production. However, difficulties in developing a stable cell line capable of high-titer LV production have meant that large-scale, transient transfection-based methods are required.

Current methods for transient LV production are based on the multi-plasmid transfection of adherent HEK 293T cells in single monolayer flasks. However, up-scaling LV production using monolayer flasks is time-consuming, labour intensive, and requires large work spaces for cell cultivation [1,14,15]. Recently, there has been a shift towards developing suspension based cultures using adapted HEK 293T cells to produce high density cultures within a limited work space by using stirred-tank and wave-bag bioreactors [16,17]. Despite some early progress, LV production in suspension conditions is still in its infancy. Hence, continued development of adherent-based LV production methods is important until the suspension culture methods are more established. Modest increases in the production capacity of adherent-cell based systems have recently been achieved by employing technologies such as roller bottles, multi-layer flasks, and cell factories [13]. Accordingly, a cell factory method was developed as an interim solution for producing LV vector for pre-clinical studies, with the potential for future large-scale production in a scale-out scenario.

**Figure 1.**
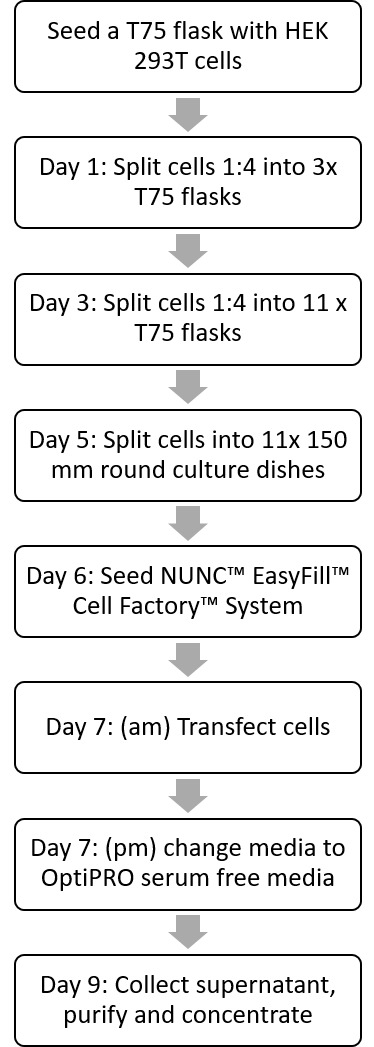
Workflow diagram of the vector production protocol. Flow diagram outlining the main steps at each day of vector production, including culturing HEK 293T cells, transfection of cells with lentiviral plasmids, changing to a serum free media and finally the purification and concentration of the final product.

Here, we describe a method to produce VSV-G pseudotyped LV vector using calcium phosphate co-precipitation in 10-layer NUNC™ EasyFill™ cell factories. In this method, downstream processing is performed *via* a Mustang Q XT5 anion exchange step followed by ultracentrifugation (**Fig. 1**). Our results show that concentrating the supernatant up to 3000 times enables us to achieve volumes of 500 µl/cell factory at a titer of 10^8^–10^9^ TU/ml (determined by RT-PCR). Although the initial expansion of cells to seed a cell factory remains time consuming, gains are made using multi-layer cell factories, as they avoid the use of multiple monolayer flasks, where one 10-layer cell factory is equivalent to 36 T175 flasks. The use of cell factories increases the surface area available for cell cultivation, thereby improving vector yields, while also decreasing the transfection and post-transfection media-change times, and reducing labor costs. Although this method describes use of a second-generation system, it is applicable to production of first, second or third generation LV vectors by choosing the appropriate plasmid ratios. It can also be used for producing LV vectors with alternative pseudotypes (*e.g.*, HA or GP64 [18,19]) with changes to the ultracentrifugation step, or for use with other transfection agents including polyethylenimine (PEI) [20].

## MATERIALS

### Cell culture

✓ HEK 293T cells (American Type Culture Collection, cat. # CRL-3216)
✓ Dulbecco’s Modified Eagle’s Medium (DMEM) (Gibco, cat. # 11965-084)
✓ Fetal calf serum (FCS) (Australian Origin) (Gibco, cat. # 10099-141)
✓ Penicillin-streptomycin (penicillin 10000 IU/ml, streptomycin 10000 µg/ml) (Gibco, cat. # 15140-122)
✓ 75 cm^2^ cell culture flasks, red filter screw cap (T75) (Greiner Bio-one, cat. # 658175)
✓ Corning tissue-culture treated culture dishes 150 mm × 25 mm (Sigma-Aldrich, cat. # CLS430599-60)
✓ Phosphate buffered saline (PBS) (Sigma-Aldrich, cat. # D8537)
✓ TrypLE express cell dissociation reagent (Gibco, cat. # 12604-021)

### Plasmid transfection

✓ NUNC™ EasyFill™ Cell Factory™ System (Thermo Fisher Scientific, cat. # 140400)
✓ Na_2_HPO_4_ (Sigma-Aldrich, cat. # S3264)
✓ HEPES (Sigma-Aldrich, cat. # H3375)
✓ NaCl (Sigma-Aldrich, cat. # S3014)
✓ CaCl_2_·2H_2_O (Sigma-Aldrich, cat. # C8106)
✓ Lentiviral packaging, envelope, and expression plasmids (*e.g.*, for our second generation, 5-plasmid system we use: pTat, pRev, pGag-Pol, pVSV-G and an expression plasmid. See **Table 1** for details). Users should produce the plasmids for their system in the appropriate quantity.
✓ 225 ml conical tubes (Falcon, cat # 352075)
✓ L-Glutamine (Gibco, cat. # 21051-024)
✓ OptiPRO™ SFM (Gibco, cat. # 12309-019)
✓ Penicillin-streptomycin (penicillin 10000 IU/ml, streptomycin 10000 µg/ml) (Gibco, cat. # 15140-122)

### Vector harvest and purification

✓ Fetal calf serum (FCS) (Australian origin) (Gibco, cat. # 10099-141)
✓ Bovine serum albumin (BSA) (Sigma-Aldrich, cat. # A7906)
✓ NaCl (Sigma-Aldrich, cat. # S3014)
✓ 0.9% saline for injection (Fresenius Kabi, cat. # AUST R 197200)
✓ Heat-inactivated serum from the species that the vector preparation is intended for (*e.g.*, mouse serum if the vector is to be delivered to mice) to minimize potential immune reaction.
✓ 500 ml centrifugation bottles (Beckman Coulter, cat. # 355607)
✓ 50 mm Polydisc AS 0.45 µm Whatman filter (GE Healthcare Life Sciences, cat. # 6724-5045)
✓ Mustang^®^ Q XT5 capsule (Pall Corporation, cat. # XT5MSTGQPM6)
✓ Masterflex peroxide-cured silicone tubing L/S 16 (Masterflex, cat. # 46400-16)
✓ Male luer lock with 3.2 mm (1/8”) hose barb connector (Cole-Parmer, cat. # EW-45505-04)
✓ Female luer lock with 3.2 mm (1/8”) hose barb connector (Cole-Parmer, cat. # EW-45500-04)
✓ 14 ml thin wall polyallomer ultracentrifuge tubes (Beckman Coulter, cat. # Z604085CA)

### Mustang Q XT5 maintenance

✓ NaOH (Sigma-Aldrich, cat. # 55881)
✓ NaCl (Sigma-Aldrich, cat. # S3014)

### Equipment

✓ Cole Parmer Masterflex^®^ L/S^®^ pump (Cole Parmer, model # 77200-50)
✓ Optima^™^ L-100 XP Ultracentrifuge with a SW40 Ti rotor (Beckman Coulter, cat. # 392050 and # 331302)
✓ Avanti^®^ J-E Centrifuge with a JA-10 rotor (Beckman Coulter, cat. # 369001 and # 369687)

### Recipes

✓ HEK 293T cell growth media: DMEM, 10% FCS, 10 units/ml Penicillin, 10 µg/ml Streptomycin
✓ 2× HeBS buffer: 1.5 mM Na_2_HPO_4_, 50 mM HEPES, 0.28 M NaCl in water, adjust pH to 7.08 and filter-sterilise
✓ 2.5 M CaCl_2_·2H_2_O in water and filter-sterilise
✓ Harvest media: OptiPRO™ SFM, 10 units/ml Penicillin, 10 µg/ml Streptomycin, 4 mM L-glutamine
✓ 2% heat inactivated serum: use serum from the species that the vector preparation is intended for to minimize potential immune reaction. Dilute serum in H_2_O and incubate at 65°C for 30 min
✓ 200 mM L-Glutamine in water and filter-sterilise
✓ 2.5% (w/v) BSA in PBS
✓ 1.5 M NaCl in water and filter sterilise
✓ Mustang Q XT5 column equilibration buffer: 1 M NaOH in water
✓ Mustang Q XT5 column storage buffer: 0.1 M NaOH and 1 M NaCl in water

## PROCEDURE

**Figure 2.**
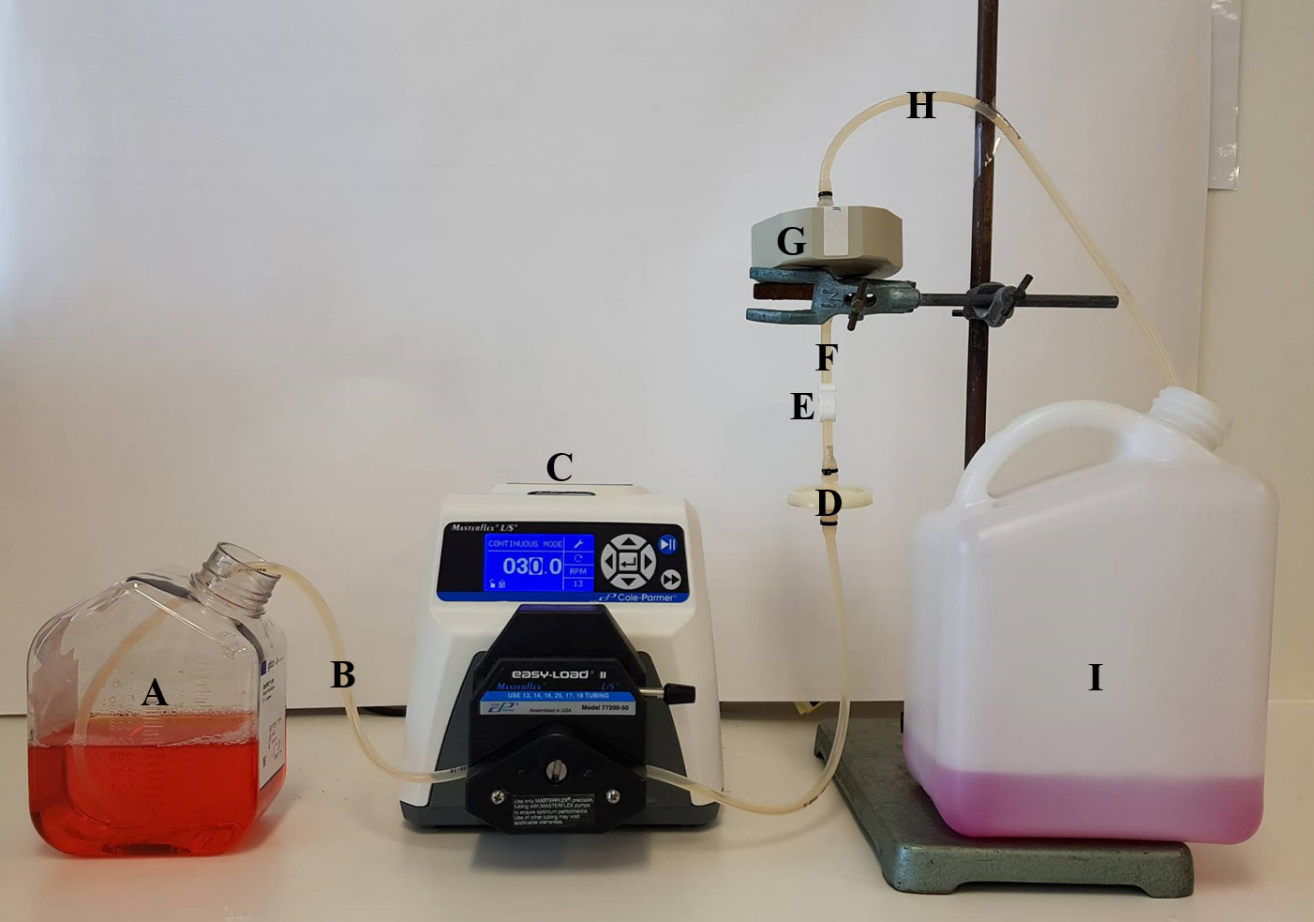
Vector purification setup. **A.** Vector supernatant obtained from cell factories. **B.** 70 cm length of MasterFlex tubing. **C.** Watson Marlow 323 pump. **D.** 50 mm Polydisc AS 0.45 µm Whatman filter. **E.** Tubing clamp. **F.** 10 cm length of MasterFlex tubing. **G.** Mustang Q XT5 column. **H.** 40 cm length of MasterFlex tubing. **I.** Waste container.

**Figure 3.**
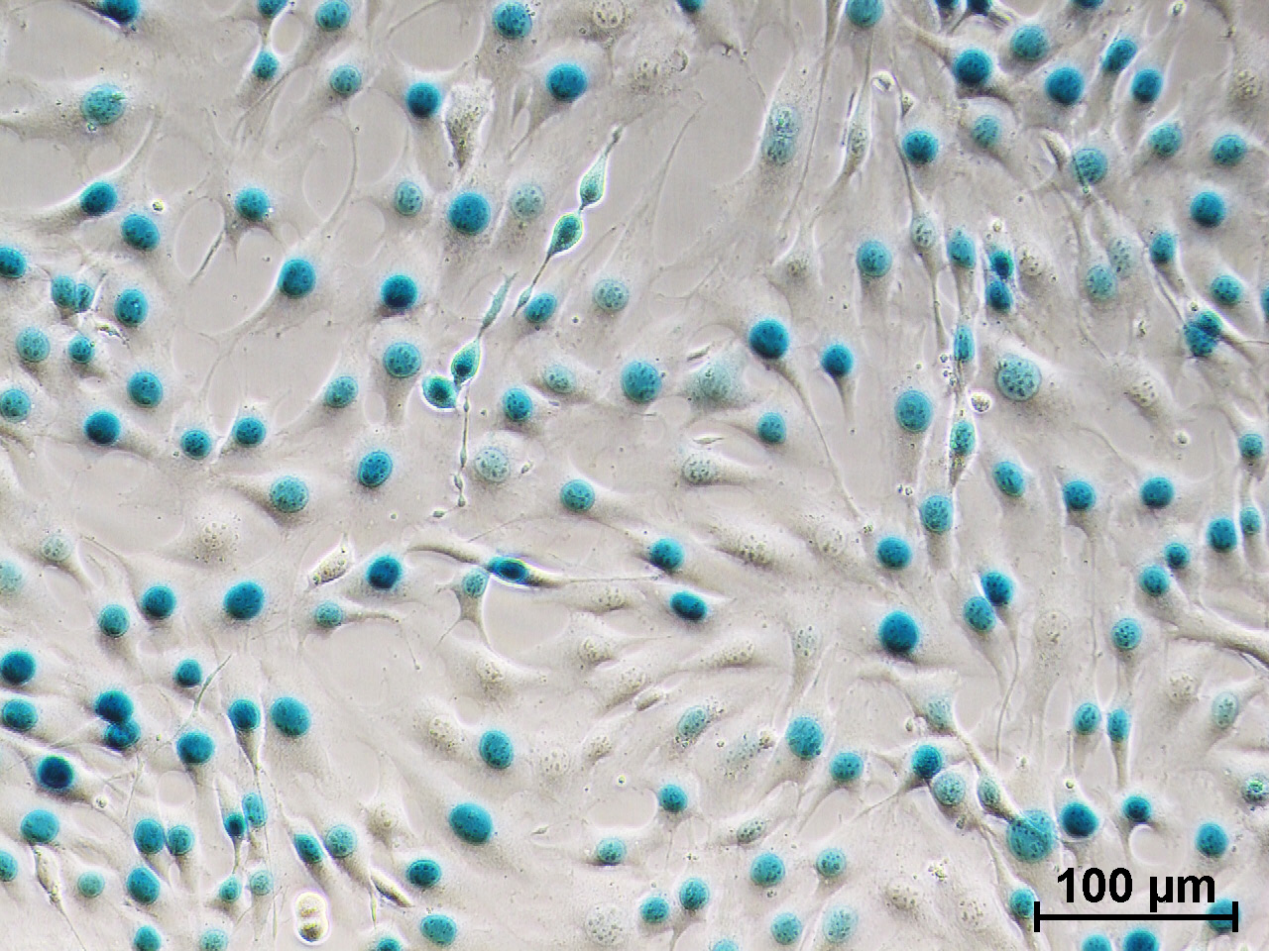
LacZ transgene expression in NIH 3T3 cells following transduction with cell factory produced LV-LacZ vector and subsequent histological X-Gal staining.

1. Culture of sufficient HEK 293T cells
  ***1.1.*** Pre-warm PBS, DMEM/10%FCS/1%Pen-Strep, and 10% TrypLE-Express in PBS to 37°C.
  ***1.2.*** Thaw a vial of frozen HEK 293T cells rapidly in a 37°C water bath and transfer to a 50 ml conical tube and make up to 50 ml with PBS. Centrifuge at 450× *g* for 5 min at 4°C. Aspirate the supernatant, re-suspend the cell pellet in DMEM/10% FCS/1% Pen-Strep and transfer to a T75 flask. Incubate at 37°C in 5% CO_2_.
  ***1.3.*** When the cells are 90% confluent, split cells (1:4) by aspirating media and washing cells with 4 ml PBS. Aspirate PBS and trypsinize cells using 4 ml of 10% TrypLE-Express in PBS, incubate for 5 min at room temperature, or until cells start to detach. Add 4 ml of media, spritz cells and transfer 2 ml to 3 × T75 flasks containing 12 ml of media.
    **NOTE:** It takes 20–24 h for HEK 293T cells to double so ensure that splitting the cells (1:2 or 1:4) is performed at the same time each morning.
  ***1.4.*** When cells are 90% confluent (approximately 48 h after seeding), repeat step two to achieve a total of 11 × T75 flasks.
  ***1.5.*** When the 11 × T75 flasks are 90% confluent, harvest cells as previously and transfer one T75 flask into one 150 mm round culture dish with a final volume of 32 ml.
2. Cell factory seeding
  ***2.1.*** Pre-warm PBS, DMEM/10%FCS/1%Pen-Strep, and 10% TrypLE-Express in PBS to 37°C.
  ***2.2.*** When the HEK 293T cells in the 150 mm round culture dishes are 90% confluent, harvest by first aspirating the media and washing cells with 6 ml of PBS. Aspirate PBS and detach cells by incubating them with 6 ml of 10% TrypLE-Express for 5 min. Add 6 ml of media to neutralize TrypLE-Express, spritz cells and transfer to a 1 L bottle.
    **TIP:** Using a left-over 1 L media bottle is a useful alternative to using multiple tubes or containers.
  ***2.3.*** Rinse each round culture dish with fresh media and add to the 1 L bottle containing the cell suspension.
  ***2.4.*** Perform a viable cell count on the cell suspension and determine the volume of cells required to achieve a final concentration of 3.476 × 10^5^ cells/ml in a total of 1500 ml. Transfer this volume to a sterile 2 L bottle and bring up to a total of 1500 ml with media.
    **CRITICAL STEP:** Using a cell density of 1.65 × 10^5^ cells /cm^2^ will ensure that the cells will reach 70%–90% confluency at 24 h post-seeding.
  ***2.5.*** Pour the cell suspension into a NUNC™ EasyFill™ Cell Factory™ System and equilibrate the chambers as per the manufacturer’s instructions.
    **TIP:** Pour the cell suspension into the large port and ensure that media enters all the layers during pouring. This will minimize the formation of bubbles within the large port that can make it difficult to transfer the full 1500 ml.
  ***2.6.*** Seed a T75 flask at a density of 6.14 × 10^6^ cells/ml to use as a reference of confluency for the cell factory. The reference flask is used to assess cell confluency because the contents of the cell factory cannot be observed under the microscope.
    **TIP:** This number of cells in a T75 ensures that the density is 1.65 × 10^5^ cells/cm^2^, which is the same as the cell factory seeding density.
3. Plasmid transfection
  ***3.1.*** We advise that users optimize the plasmid ratios for their system using small-scale preparations. Here we use example ratios for a second-generation, 5-plasmid LV vector system.
  ***3.2.*** Prepare plasmid mix (with all reagents at room temperature) in a 50 ml conical tube as per **Table 1**.
  ***3.3.*** Add the plasmid mix to a 50 ml conical tube containing 4.5 ml of 2.5 M CaCl_2_ and make up to 45 ml with sterile water.
    **CRITICAL STEP:** Ensure the pH of the 2× HeBS is exactly 7.08 as this is critical for effective DNA calcium-phosphate co-precipitation.
  ***3.4.*** Aliquot 22.5 ml of room temperature 2× HeBS into two 225 ml conical tubes.
  ***3.5.*** Begin vortexing the first 225 ml conical tube, and using a 25 ml serological pipette add 22.5 ml of plasmid solution dropwise over 30–40 s. Continue to vortex for a further 20–30 s and set aside at room temperature.
    **CRITICAL STEP:** Smaller tubes/containers will not support vortexing this large volume at a high speed without spillage. Ensure the plasmid mix is added in a dropwise fashion to maximize complex formation between plasmids and calcium phosphate.
  ***3.6.*** After a total of 90 s has elapsed from initial vortexing of the first 2× HeBS in the 225 ml conical tube, begin vortexing the second 225 ml conical tube containing 2× HeBS and again add 22.5 ml of plasmid solution dropwise over 30–40 s. Continue to vortex for a further 20–30 s and set aside.
  ***3.7.*** Retrieve the NUNC™ EasyFill™ Cell Factory™ System from the incubator, remove both caps and gently pour ~400 ml of media into a 1 L bottle *via* the small port, ensuring that cell disturbance is minimized. After four minutes has elapsed from initial vortexing of the first 225 ml conical tube, add the DNA complexes from the first 225 ml conical tube to the ~400 ml of media. After a total of five and a half minutes has elapsed, add the second 225 ml conical tube.
    **CRITICAL STEP:** This incubation period is important to aid in the plasmid-calcium phosphate complex formation.
  ***3.8.*** With care to minimize cell disruption, gently pour the media containing the plasmid solution back into the 10-layer cell factory *via* the large port, equilibrate again as per the manufacturer’s instructions, and place back in the 37°C incubator with 5% CO_2_ for eight hours.
  ***3.9.*** During the eight-hour incubation, pre-warm 1.5 L of OptiPRO SFM supplemented with 4 mM L-glutamine and 1% Pen-Strep to 37°C.
  ***3.10.*** At eight hours post-transfection, perform a media change by pouring out all of the media from the NUNC™ EasyFill™ Cell Factory™ System *via* the small port (remove as much excess media as possible) and subsequently add 1.5 L of pre-warmed OptiPRO™ SFM into the large port. Pour gently to ensure minimal disturbance to adhered cells and equilibrate media amongst the layers.
    **TIP:** Eliminating FCS from the final vector product is recommended to minimize the risk of a potential immune response to *in vivo*.
  ***3.11.*** Incubate at 37°C and 5% CO_2_ for a further 40 h.
4. LV vector harvest
  ***4.1.*** Pre-weigh 2 × 1 L bottles.
    **TIP:** Use the OptiPRO™ SFM bottles from the previous day.
  ***4.2.*** Remove the NUNC™ EasyFill™ Cell Factory™ System from the incubator and carefully pour the viral supernatant into the pre-weighed 1 L bottles.
  ***4.3.*** Add 200 ml of PBS to the large port of the NUNC™ EasyFill™ Cell Factory™ System and equilibrate to rinse any remaining viral supernatant. Pour the PBS rinse into the pre-weighed 1 L bottles.
  ***4.4.*** Weigh the bottles again to determine the volume of supernatant collected and add BSA (stored on ice) to achieve a final concentration of 0.1% BSA to the supernatant.
    **HINT:** Divide the volume of the supernatant (assuming 1 g = 1 ml) by 24 and then add that volume of 2.5% BSA (w/v) in PBS to make a final BSA concentration of 0.1%.
  ***4.5.*** Transfer the supernatant to 500 ml centrifuge bottles. Rinse each 1 L bottle with 20 ml PBS and add to 500 ml centrifuge bottles.
  ***4.6.*** Centrifuge supernatant at 400× *g* for five minutes at 4°C to remove cellular debris. Pour supernatant back into the 1 L bottles and keep refrigerated at 4°C until required.
5. Lentiviral purification and concentration
  ***5.1.*** To set up the Mustang Q XT5 column purification system (**Fig. 2**), connect a 70 cm piece of MasterFlex tubing to the Watson Marlow 323 pump. Pump PBS through the tubing at a rate of 10 ml/min, ensuring no bubbles are present within the tubing.
    **CRITICAL STEP:** It is very important to ensure no air bubbles enter the system as the presence of bubbles can cause damage to the lentivirus particles and can significantly reduce the final yield.
  ***5.2.*** Attach a 50 mm Polydisc AS 0.45 µm Whatman filter at the end of the tubing, secure with a cable-tie, and continue to flush PBS until the filter is saturated.
    **TIP:** Ensure that the inlet side of the 0.45 µm filter is facing downward so that gravity forces the supernatant to pass through the filter across the entire bed.
  ***5.3.*** Attach a 10 cm length of MasterFlex tubing with a tubing clamp to the outlet end of the 50 mm Polydisc AS 0.45 µm Whatman filter, secure with a cable-tie, and flush with PBS, ensuring no bubbles are present within the tubing.
  ***5.4.*** Attach a male M6 thread with a hose barb connector to the Mustang Q XT5 column and connect the inlet port to the 10 cm piece of MasterFlex tubing, securing with a cable-tie. Connect a 40 cm piece of MasterFlex tubing to the outlet port with a male M6 thread with a hose barb connector, securing with a cable-tie, and place the Mustang Q XT5 column in a retort stand with the outlet facing upwards. Place the end of the tubing into a large container to collect the waste.
    **TIP:** Like the filter, ensure that the outlet port of the Mustang Q column is facing up so that gravity forces the supernatant to pass through the entire surface area of the column.
  ***5.5.*** Continue to pump PBS through the system at 10 ml/min to flush out the buffer that the column is stored in. Once the pH is ~7, the system is ready to use (this can be assessed by using pH indicator strips or a pH meter).
    **CAUTION:** A pH of ~7.20 must be used for all solutions post-harvest as this is the optimal pH environment for the vector.
  ***5.6.*** Place the 70 cm length of tubing into a 1 L bottle of vector supernatant that has been stored at 4°C. Pump the supernatant through at 10 ml/min until system has been completely flushed with supernatant and then increase the pump rate to 30 ml/min.
  ***5.7.*** Once all but a few ml of the vector supernatant has been pumped through the system, rinse 1 L bottles with 150 ml of PBS containing 0.1% BSA and pump through the system.
  ***5.8.*** Turn off the pump before the 10 cm length of tubing has air introduced into it to ensure air bubbles do not enter the Mustang Q XT5 column. Close the tubing clamp and cut off the lower 7 cm of tubing. Attach a female luer fitting with a hose-barb connector to the end of the tubing (that is attached to the column), and secure with a cable-tie.
    **CAUTION:** Clamping of the tubing is important to prevent supernatant leaking from the system due to gravity.
  ***5.9.*** Using a 50 ml male luer lock syringe, flush the column with 60 ml of PBS.
  ***5.10.*** Cut off all but the last 10 cm of the 40 cm piece of tubing connected to the Mustang Q XT5 outlet port. Using a 50 ml syringe, elute the vector with 25 ml of 1.5 M NaCl (stored on ice), and capture into 25 ml of 2% heat inactivated animal serum (stored on ice).
  ***5.11.*** Transfer the elution equally into 4 × 14 ml thin wall polyallomer tubes and place into chilled SW 40 Ti buckets. Centrifuge at 20000 rpm for 90 min at 4°C.
  ***5.12.*** Following ultracentrifugation, gently pour off the supernatant, ensuring that the vector pellet is not disturbed, and blot tubes on tissue paper to remove as much residual liquid as possible.
  ***5.13.*** Resuspend the vector pellets in 0.9% saline (stored on ice) by pipetting up and down, while ensuring that no bubbles form and clumps are no longer visible. Rinse tubes to maximize vector recovery. Once resuspended, add 0.1% (v/v) heat inactivated animal serum and keep on ice when not re-suspending.
    **TIP:** To maximize vector recovery, initially resuspend vector pellets in less than a quarter of the desired final volume. Transfer this volume between each ultracentrifuge tube and finally transfer into a screw-cap 1.5 ml microcentrifuge tube. Then rinse the centrifuge tubes with a further quarter of the final resuspension volume, pool into the same 1.5 ml screw-top microcentrifuge tube and repeat with a third wash with a quarter of the final resuspension volume. Top up to the desired final volume.
  ***5.14.*** Aliquot vector into screw cap tubes and store at −80°C. Avoid freeze-thawing as this can decrease titers up to 10-fold /cycle.
  ***5.15.*** Test virus functionality using an appropriate method based on the expression plasmid used (**Fig. 3**).
6. Mustang Q XT5 column equilibration and storage
  ***6.1.*** After elution, equilibrate the column by pumping 25 ml of equilibration buffer at room temperature through the column at 10 ml/min. Stop the pump, close the tubing clamp, and allow to rest for 30 min.
  ***6.2.*** Pump through 25 ml of Mustang Q XT5 column storage buffer at room temperature at 10 ml/min, stop the pump, disconnect the column, and cap the inlet and outlet ports. Store at room temperature until next use.
    **NOTE:** This allows for long term usage of the column and will limit bacterial growth.

## TROUBLESHOOTING

Possible problems and their troubleshooting solutions are listed in **Table 2**.

## Figures and Tables

**Table 1.** Plasmid quantities used for transfection of one cell factory (based on a second generation, 5-plasmid system).

	DNA mass (μg)	DNA length (bp)	DNA copy number	Plasmid volume (μl)
Expression vector	2252.58	8986	2.32E + 14	DNA mass (μg)/plasmid concentration (μg/μl)
pcDNATat	39.98	5668	6.53E + 12	
pHCMV-Rev	39.98	5789	6.40E + 12	
pHCMV-gagpol	25.29	9771	2.40E + 12	
pVSV-G	100.08	6363	1.46E + 13	

Note: When using plasmids of different DNA lengths, it is important to maintain the same DNA copy number.

**Table 2.** Troubleshooting guide.

Step	Problem	Cases	Suggestions
2.3	Low cell yield	Cells under-confluent Culture plates not rinsed thoroughly Poor cell health	Allow cells to expand longer Rinse culture plates thoroughly to maximize cell yield Increase the number of round culture dishes Thaw a new batch of cells Check for mycoplasma contamination Do not overgrow cells (keep culture in logarithmic growth phase)
3.5	HeBS/plasmid complex solution is not cloudy	Plasmid solution added too quickly Incorrect pH of 2× HeBS Not vortexed vigorously	Plasmid solution needs to be added slowly and dropwise to 2× HeBS Ensure HeBS has a pH of 7.08 (± 0.01) Vortex as vigorously as possible
3.5	HeBS/plasmid complex solution has precipitated out	Incorrect calcium chloride used	Make sure to use Cacl_2_·2H_2_O (MW-147.01 g/mol)
5.7	Clogged filer	Too much debris in supernatant	Replace filter Centrifuge supernatant twice Use a filter with a larger binding surface area Use a series of filters with decreasing g pore size
5.14	Low tire	Poor plasmid quality Unhealthy cells Mustang Q XT5 column not equilibrated	Check quality of plasmids Ensure only healthy cells are used for production Re-equilibrate the Mustang Q XT5 column following part 6: Mustang Q XT5 column equilibration and storage Check the ultracentrifugation tubes are a suitable plastic (*e.g.*, polyallomer)
